# A novel nanoemulsion-based microalgal growth medium for enhanced biomass production

**DOI:** 10.1186/s13068-021-01960-8

**Published:** 2021-04-30

**Authors:** Harshita Nigam, Anushree Malik, Vikram Singh

**Affiliations:** 1 Applied Microbiology Laboratory, Centre for Rural Development and Technology, Hauz Khas, New Delhi 110016 India; 2 Department of Chemical Engineering, Indian Institute of Technology Delhi, Hauz Khas, New Delhi 110016 India

**Keywords:** Microalgae, Nanoemulsion, Paraffin oil, Silicone oil, Biomass

## Abstract

**Background:**

Microalgae are well-established feedstocks for applications ranging from biofuels to valuable pigments and therapeutic proteins. However, the low biomass productivity using commercially available growth mediums is a roadblock for its mass production. This work describes a strategy to boost algal biomass productivity by using an effective CO_2_ supplement.

**Results:**

In the present study, a novel nanoemulsion-based media has been tested for the growth of freshwater microalgae strain *Chlorella pyrenoidosa*. Two different nanoemulsion-based media were developed using 1% silicone oil nanoemulsion (1% SE) and 1% paraffin oil nanoemulsion (1% PE) supplemented in Blue-green 11 media (BG11). After 12 days of cultivation, biomass yield was found highest in 1% PE followed by 1% SE and control, i.e., 3.20, 2.75, and 1.03 g L^−1^, respectively. The chlorophyll-a synthesis was improved by 76% in 1% SE and 53% in 1% PE compared with control. The respective microalgal cell numbers for 1% PE, 1% SE and control measured using the cell counter were 3.00 × 10^6^, 2.40 × 10^6^, and 1.34 × 10^6^ cells mL^−1^. The effective CO_2_ absorption tendency of the emulsion was highlighted as the key mechanism for enhanced algal growth and biomass production. On the biochemical characterization of the produced biomass, it was found that the nanoemulsion-cultivated *C. pyrenoidosa* had increased lipid (1% PE = 26.80%, 1% SE = 23.60%) and carbohydrates (1% PE = 17.20%, 1% SE = 18.90%) content compared to the control (lipid = 18.05%, carbohydrates = 13.60%).

**Conclusions:**

This study describes a novel nanoemulsion which potentially acts as an effective CO_2_ supplement for microalgal growth media thereby increasing the growth of microalgal cells. Further, nanoemulsion-cultivated microalgal biomass depicts an increase in lipid and carbohydrate content. The approach provides high microalgal biomass productivity without altering morphological characteristics like cell shape and size as revealed by field emission scanning electron microscope (FESEM) images.

**Graphical abstract:**

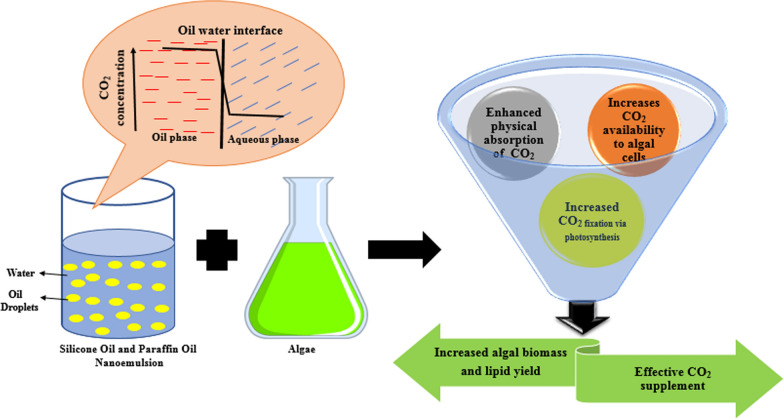

**Supplementary Information:**

The online version contains supplementary material available at 10.1186/s13068-021-01960-8.

## Background

The major bottleneck of microalgal cultivation is low biomass yield which restricts its application at a large scale [1]. In addition to environmental factors like pH, temperature, light intensity, photoperiod, composition of nutrient media plays a vital role during algal growth [2]. There have been various attempts to enhance algal biomass production and specific biomolecule content by providing algal growth supplements, phytohormones or optimizing the N/P ratio [3, 4]. Choi et al. [5] reported that the biomass productivity of *Chlorella vulgaris* (FC-16) increased from 0.78 to 2.97 g L^−1^ day^−1^ when N/P ratios were changed from 30 to 10 in the municipal wastewater. Similarly, Nahidian et al. [6] demonstrated a 32% rise in biomass productivity of *Haematococcus pluvialis* by using modified Bold’s Basal Medium (BBM) with threefold higher phosphate concentration. Renuka et al. [7] studied the effect of exogenous cytokinins on microalgae under nitrogen stress and obtained high biomass (176.79 mg L^−1^ d^−1^) as well as lipid productivity (63.14 mg L^−1^ d^−1^) of *Acutodesmus obliquus* on the addition of zeatin. The biomass and lipid productivities were enhanced by 23.4% and 60%, respectively, compared with conventional BG11.

Another major raw material required for the growth of algae is carbon dioxide. There is a growing recognition that microalgae are among the most productive biological organisms for biomass production and carbon capture [8]. The CO_2_ fixation efficiency of green algae is 10–50 times higher than terrestrial plants, be it from atmospheric air with low CO_2_ concentration or from flue gases with 12% to 15% of CO_2_ [9]. Atmospheric CO_2_ concentrations have increased from 295 parts per million (ppm) to above 400 ppm over the past 100 years, and have significantly contributed to global warming and climatic change [10]. The most environmentally sustainable way to reduce greenhouse gas emissions associated with energy production is to generate energy from carbon–neutral or reduced-carbon-emission sources like algae. CO_2_ capturing efficiencies as high as 90% have been reported in open algal ponds [11]. Furthermore, 94% CO_2_ removal efficiency was observed by algal-based airlift photobioreactor [12]. Between 1.6 and 2 g of CO_2_ is captured for every gram of algal biomass produced.

Algal cells have been observed to show high productivity at a larger concentration of carbon dioxide in the sparged gas [13]. Also, increased rates of photosynthesis are observed in elevated CO_2_ concentration in the atmosphere [14]. However, the concentration of carbon dioxide in the aqueous growth media is limited by the low solubility of carbon dioxide in water. At equilibrium with atmospheric air, water contains less than 100 ppm of free carbon dioxide [15]. To overcome this challenge of the limited supply of CO_2_ from the atmosphere to the cultivation system, most of the research has focused on the direct delivery of CO_2_ to photobioreactors, raceways, and ponds, but this approach has two main limitations. Firstly, the delivery of CO_2_ in such a manner is an energy-intensive process [16], and secondly, most of the CO_2_ escapes into the surroundings leading to loss. To overcome these limitations, chemical absorption of CO_2_ has been considered in the past, which involves the addition of solvents like carbonates and amines. Duan et al. [17], cultivated *Scenedesmus obliquus* in wastewater and obtained an increase in lipid content (from 16 to 25%) as well as biomass concentration (from ~ 0.13 to 0.21 g L^−1^) upon adding 20 mg L^−1^ sodium carbonate to the wastewater. Enhanced protein (67.1 mg L^−1^ d^−1^) and lipid (46.9 mg L^−1^ d^−1^) productivity were obtained by adding monoethanolamine during the fed-batch cultivation of *Chlorella fusca* LEB 111 although the biomass productivity was found to be similar to the control [18]. In a recent finding, solvent-based CO_2_ delivery was proposed which involves the capture of CO_2_ in the solvent and its subsequent delivery to algal cells through the non-porous membrane polydimethylsiloxane (PDMS) [16]. This membrane-based system allows the diffusion of CO_2_ from solvent into algal cultivation media, leading to significant enhancement in biomass productivity of *Chlorella* sp. from 0.021 to 0.39 g L^−1^ day^−1^. However, with this approach, the cost of membrane adds a significant capital burden for large-scale systems and also receptive to microbial contamination [19].

Therefore, an efficient CO_2_ delivery agent to the algal cultivation media is required which can be used to increase the growth, productivity, and synthesis of biomolecules inside algal cells [20]. Moreover, the CO_2_ delivery agent should be cost-effective, non-toxic, and should have a high affinity for CO_2_. Organic liquids like perfluorocarbons (PFCs) and silicone oil [21, 22] have been shown to absorb larger volumes of gases like carbon dioxide and oxygen compared to aqueous media. Emulsions of oils like PFCs in the cell growth media have been used very often to address the problem of low oxygen solubility for improving conditions for mammalian cell culture [23, 24]. However, their formulation in the form of nanoemulsion for the algal cultivation area is unexplored. The physiochemical properties like nano-size, stable suspended media, easy handling [25], and the use of ingredients (like oil and surfactant), which govern the functionality of the nanoemulsions, make it a suitable choice for the absorption of CO_2_.

In this work, we use emulsions of organic liquids to improve algal biomass production, which will boost the efficiency of algal cultivation in sequestering the CO_2_ and indirectly supports carbon neutrality in the environment. With the implementation of this technology, efficient carbon capture by biological means can be made possible. In particular, we use 1% emulsions of silicone oil and paraffin oil in the algal growth media to improve the growth of freshwater microalgae strain *Chlorella pyrenoidosa* and study its effect on microalgal cell growth, morphology, pigment synthesis, biomass yield, and biomass composition. In the next section, we discuss the methodology used in the present study for the preparation of the emulsion and conditions used for the microalgal cell culture. In “Results and discussion” section, we present a comparison of the increase in microalgal cell growth obtained in a nanoemulsion growth media with the control. We also discuss few possible mechanisms that could be contributing to the enhanced microalgal growth observed in the presence of emulsions. We finally conclude our manuscript with a future perspective emerging from our work.

## Materials and methods

### Selection of oil and surfactant

Silicone, paraffin oil, and surfactant (Tween 80) were procured from Central Drug House (New Delhi, India). Silicone oil is a nonpolar solute and has efficient gaseous absorption capacity which makes it suitable oil for our experiment. Paraffin oil is a mixture of C_10_–C_15_ alkanes and cycloalkanes which are colorless, antioxidative, and have low viscosity.

The nonionic surfactant Tween 80 (Polyoxyethylene (20) sorbitan monooleate) with the hydrophilic–lipophilic balance of 18, was used for the formulation of silicone and paraffin oil nanoemulsion. The surfactant action is to reduce the interfacial tension between two phases [26].

### Nanoemulsion formulation and characterization

The silicone and paraffin oil-in-water nanoemulsions were prepared by a previously optimized high-energy method [27]. Nanoemulsion was formulated by adding an aqueous phase in a mixture of oil and surfactant. The resulting mixture was homogenized at 10,000 rpm for ~ 15 min at 25 ºC (OV5, VELP Scientifica, Italy) and the nanoemulsion was subjected to ultrasonic emulsification by using 20 kHz EI-1000UP ultrasonicator (Electrosonic Industries, India) using a 15-mm titanium probe; the sonicator having sequential cycles of 45 min, with sequential on and off.

The resultant nanoemulsion was visually observed, to analyze the change in physical appearance. Nearly 25 mL of silicone and paraffin oil nanoemulsion kept undisturbed in an 80-mL beaker at 25 ºC were observed for sedimentation or creaming for 15 days. The average diameter of the droplet size of silicone and paraffin oil nanoemulsion was observed by dynamic light scattering (DLS) by photon-associated spectrometer Malvern 4700 zeta-sizer (Anton Paar, Austria) equipped with an argon laser at a wavelength of 488 nm. The temperature of the nanoemulsions was maintained at 25 ºC and the scattering angle was fixed at 90º. The nanoemulsions were withdrawn on the 0th day and 15th day and observed for the DLS study. The determination of the droplet size of silicone oil and paraffin oil nanoemulsions was carried out by diluting 1 mL of the nanoemulsion with 10 mL of water to avoid multiple scattering.

### CO_2_ measurement set-up and analysis

**Fig. 1:**
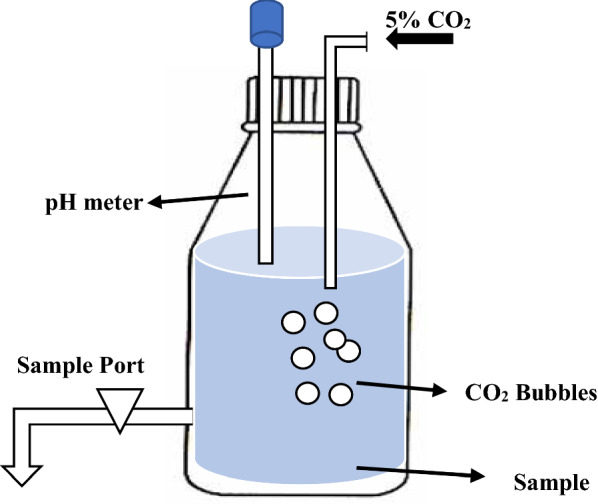
A schematic representation of dissolved free CO_2_ analysis

In the present study, we determined the availability of free carbon dioxide by titration with standard sodium hydroxide solution. The schematic of the apparatus shown in Fig. 1 was used for dissolved free CO_2_ measurement in nanoemulsions and deionized water. CO_2_ solubility and speciation in water are governed by the instantaneous pH of the system with free CO_2_ and carbonic acid forming major constituents as we go below pH 7 [3]. A gas stream containing 5% CO_2_ balanced with nitrogen was introduced in the glass bottle that contained nanoemulsion by controlled sparging using a precise gas flow regulator for 10 min duration. The pH of the nanoemulsion was monitored continuously during sparging using a pH probe dipped inside the glass bottle. After sparging the gas, the sample of nanoemulsion was taken for titration. Deionized water (DI) was used as control. The experiments were performed in triplicates and free CO_2_ concentration was measured at intervals of 60 min for 6 h.

The dissolved free CO_2_ concentration (DCC) in the nanoemulsions was determined by the titrimetric American Public Health Association (APHA) standard protocol [28]. The nanoemulsions were titrated with NaOH solution (0.01 N) using phenolphthalein as an indicator until a light pink color change was observed. All titrations were performed at room temperature with manual stirring. The DCC was determined using the equation:

1 $$C_{{CO_{2} }} \left( {mg L^{ - 1} } \right) = \frac{{\left[ {{\text{Vol}}.{\text{ of NaOH }} \times {\text{Conc}}.{\text{ of NaOH }}\left( {\text{in normality}} \right) \times 1000 \times {\text{Eq}}.{\text{ Wt of CO}}_{2} } \right]}}{{\text{Volume of the sample }}},$$

where, Eq. Wt is the equivalent weight of CO_2_, i.e., 22.

### Microalgae strain and inoculum preparation

Pure culture of microalgal species *Chlorella pyrenoidosa* was previously procured from the National Collection of Industrial Microorganisms (NCIM), NCL Pune (India). Blue Green 11 broth of HiMedia M1541 was used as standard algal growth media. The algal culture was maintained in the algal growth chamber under LED light source ~ 46.5 to 50 µmol m^−2^ s^−1^ to provide the required photon flux density for photosynthesis of microalgal cultures with light and dark cycle of 12:12 h. The incubation temperature of microalgal cultivation was 25 ± 1 ºC.

The inoculum of pure microalgal strain *C. pyrenoidosa* was cultured in 100 mL BG 11 media using 250-mL Erlenmeyer flasks. The flasks were incubated in an algal growth chamber for enrichment under light intensity ~ 46.5 to 50 µmol m^−2^ s^−1^ for 12–15 days at 25 ± 1 °C. During the log phase of the growth cycle, 30% (v/v) of microalgal suspension from stock cultures (optical density ~ 2.0 at 680 nm) was used as inoculum for experimental sets [29].

### Experimental design

The developed 100 mL of 1% SE and 1% PE nanoemulsion was mixed with 0.16 g BG11 growth media and inoculated with *C. pyrenoidosa*. Control that consisted of only BG11 media without 1% SE and 1% PE nanoemulsion was also inoculated with *C. pyrenoidosa* in BG11. The flasks capped with cotton plugs were incubated under controlled conditions with light intensity ~ 46.5 to 50 µmol m^−2^ s^−1^, dark:light regimen of 12:12 h, temperature: 25 ± 1 °C, and shaking at 150 rpm [30]. No CO_2_ sparging was done during this experiment. The time duration of the experiments was 12 days. Samples were drawn at every 2-day interval to monitor the growth of *C. pyrenoidosa* by measuring optical density at 680 nm, chlorophyll-a, and pH. Additionally, the viability testing of microalgal cells was performed via 3-[4,5-dimethylthiazol-2-yl]-2,5-diphenyl tetrazolium bromide (MTT) assay and SYTOX green staining on the 8th day of the experiment. On day 12, the samples were drawn, and cell count measurements were done. After the completion of the experiment on the 12th day, algal biomass was harvested using centrifugation for estimation of biomass yield and composition. Further, the harvested microalgal biomass from different sets was characterized by field emission scanning electron microscope and Fourier transform infrared spectroscopy.

### Analytical techniques

#### Microalgal growth analysis in formulated nanoemulsions

The optical density of *C. pyrenoidosa* was measured at 680 nm by using UV–Vis spectrophotometer (Lambda 35, Perkin Elmer, USA), and a corresponding blank with BG11 media without algae was used as the control [31]. The aliquots of the 2-mL microalgal sample were withdrawn from well-mixed microalgal culture from the experimental unit and centrifuged at 3600 g for 5 min. The obtained pellets were washed three-four times by 0.15 M phosphate buffer saline (pH 6.8) and surfactant (Tween 80) to remove silicone and paraffin oil nanoemulsions. The resultant pellets were resuspended in 2 mL distilled water and placed in the cuvette for observation. The pH of microalgal cells cultivated in nanoemulsions was measured by pH meter (10BN, Eutech, US) at a regular interval of 48 h.

Chlorophyll-a estimation was performed by the method suggested by Chinnasamy et al. [32]. 2 mL of microalgal cell suspension was collected in microcentrifuge tubes and centrifuged at 3600*g* for 10 min. The pellet formed was resuspended in 2 mL of methanol after decanting and placed in the water bath for chlorophyll extraction at 60 ºC for ~ 30 min. The chlorophyll content in the supernatant was observed spectrophotometrically at 750, 665.2, and 652 nm, and then calculated by Porra’s equation [33]:

2 $${\text{Chl a }}\left( {\mu {\text{g mL}}^{{ - {1}}} } \right) \, = { 16}.{29 }\left( {{\text{A}}^{{{665}.{2}}} - {\text{A}}^{{{75}0}} } \right) \, - { 8}.{54 }\left( {{\text{A}}^{{{652}}} - {\text{A}}^{{{75}0}} } \right),$$

where, A^750^, A^665.2^, and A^652^ are the absorbance of the chlorophyll in methanol, respectively.

Harvested algal biomass was used to determine the biomass yield (dry cell weight) on the 12th day. The 50-mL algal suspensions were centrifuged (R-8C, REMI, INDIA) at 3600 g for 10 min and then the supernatants were decanted. The pellets were washed as mentioned previously. The remaining pellets were suspended in distilled water (volume made up to 50 mL) and filtered with pre-weighed Glass Microfiber Filters”, GFC (Whatman). The filters with microalgal biomass were subjected to oven-drying at 60 ºC for 24 h and then cooled at room temperature in a desiccator and weighed. The net dry weight was calculated by the following formula:

3 $${\text{Biomass yield}} \left( {g L^{ - 1 } } \right) = \frac{{\left( {W_{a} - W} \right)}}{V} \times 1000,$$

where Wa and W are the weight of dried algal biomass with filter paper and weight of dried filter paper, respectively, and V is the volume of the sample taken.

The biomass productivity (g L^−1^ d^−1^) was evaluated according to the following equation [34]:

4 $${\text{Biomass productivity}} = \frac{{\left( {W_{f} - W_{i} } \right)}}{\Delta t},$$

where *Wi* and *W*_*f*_ are the initial and final concentration of microalgal biomass yield, respectively, and Δt is the time of cultivation in days.

Additionally, cell counting was performed by using cellometer (Catalog No. CHT4-PD100, Nexcelom Bioscience LLC) to evaluate the total cell number on 12th day.

#### MTT assay and SYTOX^®^ green staining

To determine the viability of microalgal cells, 3-[4,5-dimethylthiazol-2-yl]-2,5-diphenyltetrazolium bromide (MTT) assay was performed. 5 mg mL^−1^ of MTT dye was formed by dissolving in PBS. The microalgal pellets from the 2-mL culture were washed with PBS, followed by the addition of MTT dye (final concentration of 0.5 mg mL^−1^) and incubation at 37 ºC for 4 h. After the removal of the MTT dye solution from microalgal cells by centrifugation, dimethyl sulfoxide, and isopropanol (1:1) were added. The result was observed by measuring absorbance at 570 nm [35].

Additionally, to check the viability of *C. pyrenoidosa* cells, the microalgal cells were stained with nucleic acid stain SYTOX^®^ green (Cat. No. S7020, Invitrogen). The Sytox stock solution of 50 µM was prepared and 5 µL of stock solution was added to 95 µL microalgal samples, resulting in a final dye concentration of 5 µM [36]. Microalgal cells were incubated at room temperature in the dark for 30 min. The dual fluorescence mode was used to observe the stained microalgal cells. The red autofluorescence showed live cells and green fluorescence represents dead microalgal cells (excitation/emission: 504/523 nm) [37].

#### Fourier transform infrared spectroscopy (FTIR)

A known volume of suspension was withdrawn and centrifuged at 3600*g* for 10 min. The pellets were washed 2–3 times with PBS of 0.15 M and placed for lyophilization (Allied Frost FD3). The dried microalgal powder was ground and homogenized with KBr at the ratio of 1:100, and the mixture was pressed by a molding machine with a pressure load of 200 kg cm^−2^ for 5 min [38]. The fine KBr pellet which consists of dried microalgae pwder was prepared and placed at the deuterated triglycine sulfate (DTGS) detector of Nicolet Is50 (Thermo Scientific) FT-IR spectrometer. The transmittance spectra were collected between 400 to 4000 cm^−1^.

#### Field-emission scanning electron microscopy (FESEM)

Surface morphology and cell size were detected by Field Emission Scanning Electron Microscopy (FESEM, JSM-7800F Prime, Jeol). The microalgal suspension was centrifuged at 3600*g* for 5–10 min. The resultant pellet was washed 2–3 times with 0.15 M PBS. After washing, the pellet was fixed using the fixative solution (2.5% glutaraldehyde) for 30 min. The resultant pellets were washed in PBS 3–4 times followed by serial dehydration with 25%, 50%, 75%, 90%, and 100% ethanol, respectively. Finally, the fixed cells were dried at room temperature and coated with platinum [39].

### Biochemical composition of microalgal biomass

After harvesting, the microalgal biomass was dried at 65–70 ºC for 48 h for biochemical compositional analysis (till a constant mass is reached). Phenol–sulfuric acid was used for the analysis of total carbohydrate content [40]. Dried algal biomass (100 mg) was hydrolyzed with 2.5 N HCl and placed in a water bath for 3 h at 100 ºC, followed by cooling at room temperature and neutralized with powdered sodium carbonate. After neutralization, 0.1 mL sample was pipetted out in a fresh tube and diluted to 1 mL with water. After dilution, 1 mL phenol and 5 mL sulfuric acid were mixed and cooled in the water bath at 25–30 ºC. The absorbance of the sample was measured at 490 nm and the total carbohydrate was calculated using the standard calibration curve of glucose (Additional file 1: Fig. S1).

For estimation of proteins, 50 mg of dried algal biomass was taken and resuspended in 2 mL of 1 N sodium hydroxide. The mixture was incubated at 80 °C for 1 h for extraction of protein from biomass followed by cooling. Further, the mixture was centrifuged at 8960*g* at 20 °C for 20 min. The obtained supernatant was neutralized using 6 N HCl. The mixture was used for the estimation of total protein content by using the Folin–Lowry method [41].

Total lipid content was determined by modified Bligh and Dryer’s method [42]. The dry microalgal biomass (0.5 g) was blended with 100 mL of distilled water and the mixture was kept in the microwave oven at 100 °C for 5 min for cell wall rupturing. The total lipid content was then extracted by mixing chloroform–methanol mixture (1:1 v/v) with the microalgal mixture in the proportion of 1:1. The mixture was kept for separation and the lower solvent phase was removed. The removed solvent phase was filtered with Whatman filter paper and the filtrate was stored in a vial. An additional 5 mL of chloroform was mixed with the remaining microalgal pellet and the aqueous phase and homogenized for 2 min. The obtained mixture was again filtered through Whatman filter paper and then added to the former filtrate. The filtrate was placed in the graduated cylinder and the volume of the chloroform layer was noted. 0.5-mL aliquots of the chloroform layer were placed overnight into pre-weighed aluminum pans for evaporation. The lipids were estimated gravimetrically by recording the weights and then converting them into percent lipids.

Additionally, the lipid productivity (g L^−1^ day^−1^) was calculated by using the formula,

5 $${\text{Lipid}}\;{\text{ productivity}}\; = \;{\text{biomass }}\;{\text{productivity}}\; \times \;{\text{lipid}}\;{\text{ content }}\left( \% \right).$$

### Statistical analysis

All data graphs were plotted using Origin 8.5 software. To ensure the reliability of the experimental data, all analytical procedures have been performed in duplicates, and the results are expressed as mean ± SD (standard deviation). The results were analyzed by the Student’s *t*-test to identify the significant difference between control and experiment groups, wherever applicable. The *p*-value less than 0.05 is considered statistically significant while the *p*-value less than 0.005 is considered statistically very significant.

## Results and discussion

### Nanoemulsion stability analysis

**Fig. 2:**
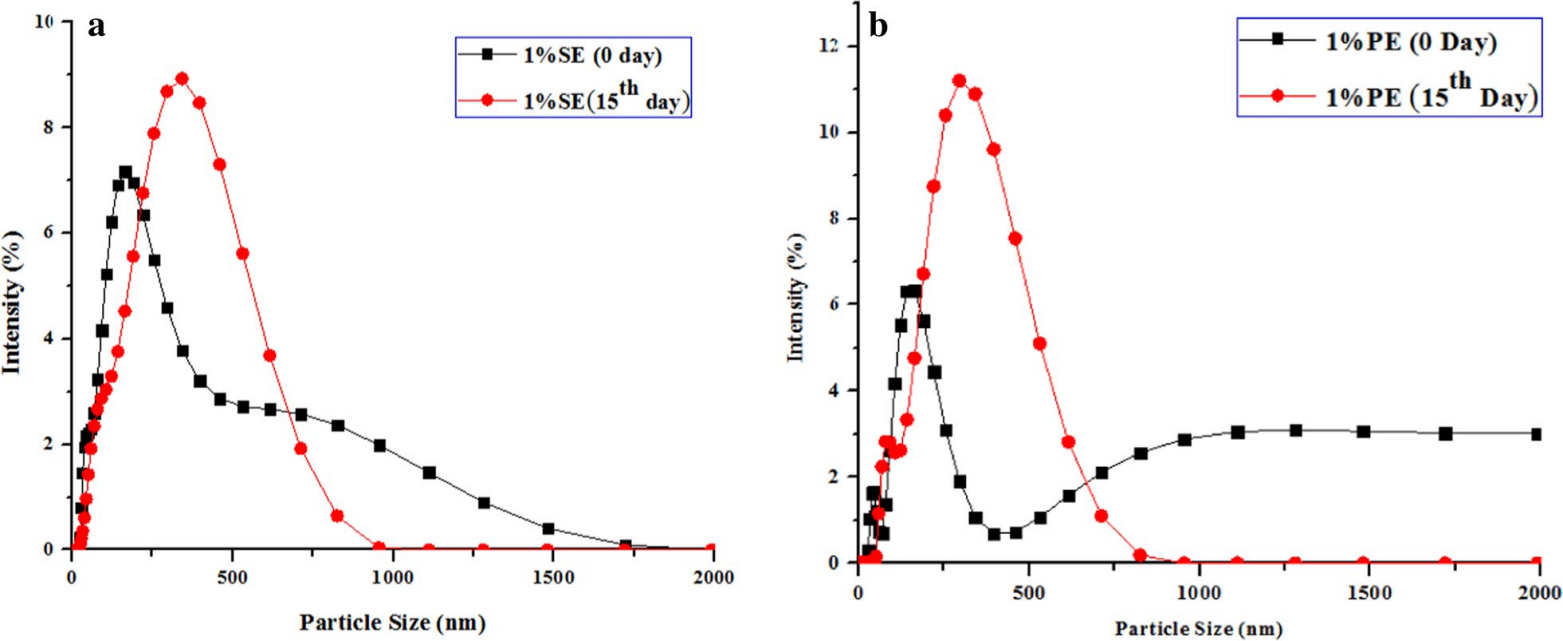
Comparison of droplet size distribution obtained from DLS for (**a**) 1% silicone oil nanoemulsion (1% SE) on 0th day and 15th day and (**b**) 1% paraffin oil nanoemulsion (1% PE) on 0th day and 15th day

The stability of nanoemulsion was observed in terms of their appearance and droplet size [43]. The samples were stored undisturbed for 15 days at 25 ºC for observation. On visual inspection, there was no creaming and flocculation in the nanoemulsion observed during the storage period. Additionally, the stability of the nanoemulsions was evaluated by droplet size measurement. A rapid increase in droplet size corresponds to an unstable nanoemulsion. Figure 2 displays the droplet size of 1% SE and 1% PE on the 0th day and 15th day, respectively. The average droplet size observed in 1% SE was in the range of ~ 244 nm to 285 nm and ~ 164 nm to 297 nm in 1% PE. As evident from Fig. 2, the size of the maximum droplets of nanoemulsions was stable over a period of 15 days. The result indicated that the larger droplet size, greater than a micron has been separated from the emulsion.

### Analysis of dissolved free CO_2_ concentration in nanoemulsions

To determine the CO_2_ uptake efficiency in nanoemulsions, the CO_2_ absorption experiment was carried out using 1% SE, 1% PE and deionized water. In all cases, the DCC value increased with time and reached saturation within 1 h. The results depicted that 1% SE retained the maximum amount of CO_2_ followed by 1% PE and DI water. The pH dropped relatively fast in DI water than nanoemulsions with the addition of CO_2_ and the final DCC observed in DI water was 8.8 mg L^−1^, due to the absence of ions. On the other hand, in nanoemulsions pH dropped slowly and the final DCC observed was 14.1 mg L^−1^ in 1% SE and 13.2 mg L^−1^ in 1% PE, which suggested that an additional amount of CO_2_ was retained in nanoemulsions compared with DI. The reason for increased CO_2_ concentration in 1% SE and 1% PE might be the presence of the organic phase (i.e., oil) in the nanoemulsions which absorbed additional CO_2_ as the partial pressure of CO_2_ during sparging was higher. Based on the obtained result, it can be interpreted that 1% SE and 1% PE retained 60% and 50% higher free CO_2_ compared with DI water.

### Growth of microalgae in nanoemulsions

In the present study, 1% SE and 1% PE were prepared to test its potential in microalgal cultivation. Cell growth in formulated nanoemulsions was evaluated by the following parameters: optical density at 680 nm, pH, Chl-a, biomass yield, biomass productivity, and cell count.

Interestingly, for both the nanoemulsions, *C. pyrenoidosa* showed an increase in growth compared with conventional BG11 media (control). Results obtained from measuring OD _680_, showed that ~ 1.6-fold growth was observed in 1% SE supplemented growth medium compared with control on the 10th day. On the other hand, 1% PE growth medium gave an increment of ~ 1.8-fold in microalgal cell growth compared with control. In 1% SE and 1% PE supplemented microalgal cultivation systems, the accelerated growth phase was observed from day-1 compared with control. This prolonged growth phase lasted till the 10th day of the microalgal cultivation in nanoemulsions while in the control lasted till the 8th day.

**Fig. 3:**
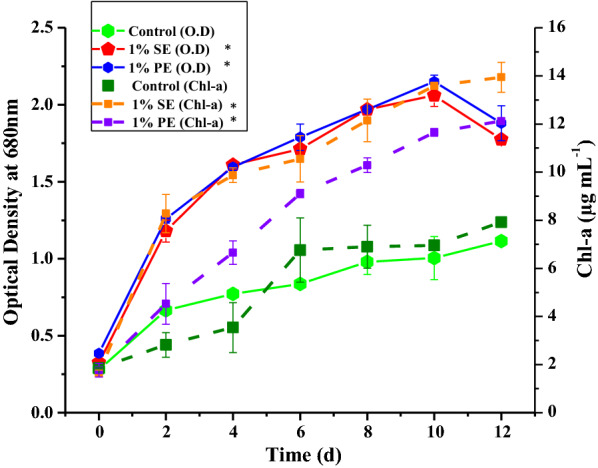
Growth profile and pigment synthesis of *C. pyrenoidosa* cultivated in 1% silicone oil nanoemulsion (1% SE) and 1% paraffin oil nanoemulsion (1% PE) compared with control (BG11) in terms of OD_680_ and Chl-a content. Solid lines in the figure represent optical density and broken lines display Chl-a synthesis. The microalgal growth and pigment synthesis were measured every 48 h up to 12 days. The cultures were operated at 25 ± 1 °C with ~ 46.5 to 50 μmol m^−2^ s^−1^ light intensity for 12 days. The data shown are the average of two data points, and error bars represent standard deviation. Data followed by an asterisk (*) are significantly different from control (*p* < 0.005, analyzed by t-test)

Gonçalveset al. [44] reported that increased CO_2_ concentration in the microalgal cultivation system extends the exponential phase. Araujo et al. [45] observed that the addition of CO_2_ during cultivating of diatom (*Chaetoceros cf. wighamii*) protracted the exponential phase, and this phase of the life span of microalgae has the highest nutritional value when used for aquatic animals as feed. In the present study, the growth pattern including the duration of the prolonged growth phase was quite similar for both the nanoemulsions. The microalgal cell growth during the accelerated phase was much higher in nanoemulsion systems compared with control (Fig. 3).

The increase in microalgal growth in nanoemulsions was probably due to the increased CO_2_ captured by the nanoemulsions from the atmosphere compared with control as discussed in the “Analysis of dissolved free CO_2_ concentration in nanoemulsions” section. The higher CO_2_ content in nanoemulsions was supported by the pH measurement of nanoemulsion-substituted microalgal cultures. The change in pH observed in control (~ 41%) was comparatively lower than the change in pH observed in 1% SE (~ 89%) and 1% PE (~ 90%). The increase in pH was observed in cultivation with 1% PE (~ 5.4 to 10.3) and 1% SE (~ 5.2 to 9.9) nanoemulsion, which was probably due to an increase in hydroxyl ion concentrations during uptake of bicarbonates and CO_3_^2−^. In the case of 1% PE-substituted algal cultivation, the CO_3_^2−^ could be the dominant species which probably increased the pH value above 10. The higher pH change suggests that more microalgal cells were replicated in nanoemulsion-substituted media compared with control as the rise in pH of cultivation media directly corresponds to the algal growth [46]. This study suggested that the 1% SE and 1% PE act as suitable alkalescent culture media which was appropriate for the growth of microalgae [47].

Furthermore, pigment synthesis in terms of Chl-a was investigated in *C. pyrenoidosa* cultivated in 1% SE, 1% PE and control as a function of culture time. It was found that 1% SE and 1% PE enhanced Chl-a pigment synthesis by 76% and 53% compared with control (Fig. 3). The increase in Chl-a concentrations in nanoemulsions cultivated biomass could be due to the sufficient availability of CO_2_ to the microalgal cells. Additionally, it might be possible that small oil droplets of nanoemulsion increase the residence time of light inside nanoemulsion leading to enhanced availability of light and enhanced pigment synthesis by microalgal cells (explained in 3.6). Besides, a slight decrease (~ 15%) in Chl-a content was observed in microalgal cells cultivated in 1% PE compared to 1% SE. This change is directly linked with the rise in pH during microalgal cultivation. Increased pH results in the volatilization of ammonia [48]. The volatilization of ammonia affects the nitrogen metabolism of algal cells and creates nitrogen-limiting conditions, thereby impacting the chlorophyll content in microalgae [49]. Literature also suggests that the increase in CO_2_ concentration leads to acidification of the algal cultivation system. This results in the replacement of Mg^+2^ with H^+^ and the formation of pheophytin instead of chlorophyll [50]. Salehi et al., [51] studied the effect of n-alkane on *Chlorella vulgaris* and reported that the existence of hydrocarbons in the medium increases the permeability of the cell wall, resulting in the accumulation of hydrocarbons inside the algal cell. This might be the reason for the inconsistency between chlorophyll concentration and cell concentration in the case of 1% PE because n-alkanes are the major portion of paraffin oil.

Hence, overall it can be summarized that 1% SE and 1% PE nanoemulsions act as active CO_2_ carriers which promote microalgal growth and Chl-a synthesis compared with conventional growth media (BG11).

**Fig. 4:**
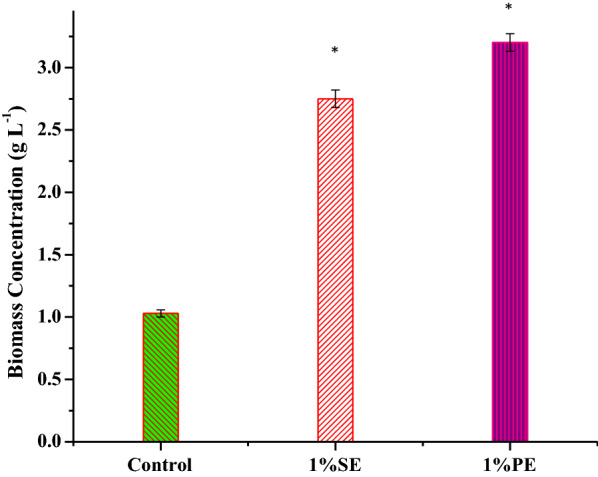
Growth profile of C. *pyrenoidosa* was observed in terms of biomass yield (g L^−1^) in 1% silicone oil nanoemulsion (1% SE), 1% paraffin oil nanoemulsion (1% PE), and control (BG11). The cultures were operated at 25 ± 1 °C with ~ 46.5 to 50 μmol m^−2^ s^−1^ light intensity for 12 days. The data shown are the average of two data points, and error bars represent standard deviation. Data followed by an asterisk (*) are significantly different from control (*p* < 0.005, analyzed by *t*-test)

The biomass yield of *C. pyrenoidosa* obtained in both the nanoemulsions was higher compared with control (~ 1.03 g L^−1^) (Fig. 4). Comparing both the nanoemulsions, the biomass yield of *C. pyrenoidosa* was higher in 1% PE (~ 3.20 g L^−1^) than 1% SE (~ 2.75 g L^−1^). Table 1 highlights the biomass yield of *Chlorella* sp*.* obtained using different growth media by various researchers. Out of these, Chi et al. [52] obtained maximum biomass yield of *Chlorella* sp*.* was about 1.10 g L^−1^ in Bold’s Basal media. Chandra et al. [53] reported maximum biomass yield of *Chlorella minutissima* was 1.84 g L^−1^ while cultivating in a modified CHU-13 medium by varying different abiotic factors. Additionally, Choix et al. [54] studied biomass yield of *Chlorella vulgari*s U162, *Chlorella* sp., *Scenedesmus obliquus* U169, and *Scenedesmus* sp. by filtered tequila vinasses and bio-digested as cultivation media. The maximum biomass yield was recorded as 2.30 g L^−1^ obtained from *Chlorella* sp. cultured in tequila vinasses. The biomass concentration of *Chlorella sorokiniana* was recorded at 1.00 g L^−1^ while using CO_2_ from flue gas as a carbon source [55]. Wong [56] used five different media to grow *Chlorella* sp*.* and got a maximum biomass concentration of 1.42 g L^−1^ using Bold’s Basal Media. In another such study, Prajapati et al. [40] found the biomass yield 0.98 g L^−1^ using Tap media. To efficiently deliver CO_2_ to algal cells, Zheng et al. [57] used a membrane-based system and found an increase in biomass concentration and the maximum biomass yield achieved was 1.77 g L^−1^ with CO_2_ loading. In another study, *Spirulina* sp. LEB 18 was cultivated in the Zarrouk medium with a mixture of diethanolamine (1.64 mmol L^−1^) and potassium carbonate (0.41 mmol L^−1^). The combination of diethanolamine and potassium carbonate enhanced the dissolved inorganic carbon concentration in the cultivation medium resulting in an increased biomass concentration of 2.10 g L^−1^ [58]. Interestingly, the biomass yield in 1% SE and 1% PE was higher compared to all the reported studies so far, signifying the importance of this cultivation media.

**Table 1:** Comparison of biomass yield of *Chlorella* sp. in different cultivation media

Growth medium	Biomass yield (g L^−1^)	References
Bold’s Basal Media	1.10	[52]
Modified CHU-13	1.84	[53]
Tequila vinasses	2.30	[54]
BG11 + CO_2_ from flue gas	1.00	[55]
Bold’s Basal, modified BG-11	1.42 ± 0.01, 0.90 ± 0.01	[56]
Carbonate-based media at 0.5 and 0.7 loading concentration	1.63 ± 0.10 and 1.77 ± 0.02	[16]
Zarrouk medium + mixture of diethanolamine and potassium carbonate	2.10	[58]
Urea + K_2_HPO_4_ + MgSO_4_·7H_2_O and ammonium ferric citrate	1.37	[103]
Tap water	0.98 ± 0.11	[40]
1% SE and 1% PE amended with BG-11	2.75 ± 0.07 and 3.20 ± 0.07	Present study

Correspondingly, the biomass productivity was evaluated after the 12th day. The biomass productivity of *C. pyrenoidosa* in 1% PE and 1% SE was 0.26 and 0.22 g L^−1^ day^−1^, respectively. The biomass productivity of *C. pyrenoidosa* was found 0.08 g L^−1^ day^−1^ in control which was lower than 1% PE and 1% SE (Table 2). Yu et al. [59] obtained biomass productivity (0.24 g L^−1^ day^−1^) of *Chlorella vulgaris* in a mixotrophic mode of cultivation. Chen et al. [60] reported 0.31 g L^−1^ day^−1^ biomass productivity of *Chlorella* sp*.* C2 in the presence of nutrient-rich ash and flue gas. Similarly, the periodic addition of monoethanolamine and CO_2_ during the cultivation of *Chlorella fusca* LEB 111 yield biomass productivity of 0.15 g L^−1^ day^−1^ [18]. *Chlorella* sp.T12 was cultivated in four different cultivation media (NSIII, Chu No. 10, BG11, and BBM) and obtained the highest biomass productivity (0.20 g L^−1^ day^−1^) in BBM media [61]. Furthermore, the productivity of *Chlorella fusca* LEB 111 was reported 0.14 g L^−1^ day^−1^ cultivated in the presence of a magnetic field [62]. In another study, *Chlorella vulgaris* was cultured in seawater media and obtained 0.13 g L^−1^ day^−1^ biomass productivity in presence of the following: 2% CO_2_, light intensity 10,000 lx, 0.3% Walne nutrient concentration and, 7 days of cultivation period [63]. Similarly, Shabani et al. [64] cultivated *Chlorella vulgaris* in various levels of salinity and CO_2_ concentration and found biomass productivity of 0.09 g L^−1^ day^−1^ in natural water at 10% CO_2_ on the 8th day. The marine microalgal strain *Nannochloropsis* sp. was cultivated in presence of CO_2_ delivered through semipermeable membranes along with chemical solvent with biomass productivity of 0.10 g L^−1^ day^−1^ [65]. Biomass productivity of *C. pyrenoidosa* obtained in both the nanoemulsions in the present study was significantly higher than the reported results.

**Table 2:** Comparison of biomass productivities of *Chlorella* sp. in different cultivation media

Growth medium	Biomass productivity (g L^−1^ d^−1^)	References
BG11 + monoethanolamine + CO_2_	0.15	[18]
BG11 + CO_2_ + acetic acid	0.24	[59]
BPPA2	0.31	[60]
Bold’s Basal Media	0.20	[61]
BG11	0.14	[62]
Seawater media	0.13	[63]
Bold’s Basal Media + natural water + CO_2_	0.09	[64]
1% SE and 1% PE amended with BG-11	0.22 ± 0.07 and 0.26 ± 0.07	Present study

The better CO_2_ absorption in the organic phase of nanoemulsions could have resulted in high microalgal biomass productivity. A similar study was performed by Sawdon and Peng [66] using perfluorocarbon emulsion for the cultivation of *C*. *vulgaris* in tubular photobioreactor. They observed a fourfold increase in microalgal cell concentration, because of efficient CO_2_ delivery to microalgal cells by perfluorocarbon emulsion. Apart from this, silicone oil had also been studied in the delivery of respiratory gases (CO_2_ and O_2_) in microbial cultivation [67], mammalian cell culturing [68], and in human retinal treatment [69].

To further cross-confirm the above results, direct cell counting was performed for observing the microalgal cell population in 1% SE, 1% PE, and control on the 12th day of the experiment using a cell counter. Result showed that 1% PE had the maximum cell number (3.0 ± 0.21 × 10^6^ cells mL^−1^), followed by 1% SE (2.4 ± 0.30 × 10^6^ cells mL^−1^) and control (1.34 ± 0.09 × 10^6^ cells mL^−1^). In a similar such study where *Chlorella vulgaris* was cultivated with 8% CO_2_ concentration, a cell count of 1.3 × 10^7^ cells mL^−1^ was obtained on the 10th day [70]. In another study during the cultivation time of 20 days, *Chlorella sorokiniana* gave cell density of 5.82 ± 0.03 × 10^6^ cells mL^−1^ [71]. However, an exact comparison cannot be made as the strain type, cultivation media, and growth conditions vary in the reported studies. In the present study, the obtained cell numbers were in agreement with the cell growth and biomass yield obtained from 1% PE and 1% SE nanoemulsions. Hence, from the present study, it was concluded that the 1% SE and 1% PE have successfully enhanced the potential of BG11 media by acting as an efficient growth supplement for microalgal cells. However, as mentioned in “Analysis of dissolved free CO_2_ concentration in nanoemulsions” section, the DCC of 1% SE was higher than the 1% PE but the microalgal growth, cell count and biomass yield obtained was higher in 1% PE supplemented microalgal cultivation. This could be due to the chemical composition of paraffin oil and the most probable explanations were highlighted in “Other possible mechanisms for improved algal growth in nanoemulsions” section.

### MTT, SYTOX^®^ green staining

**Fig. 5:**
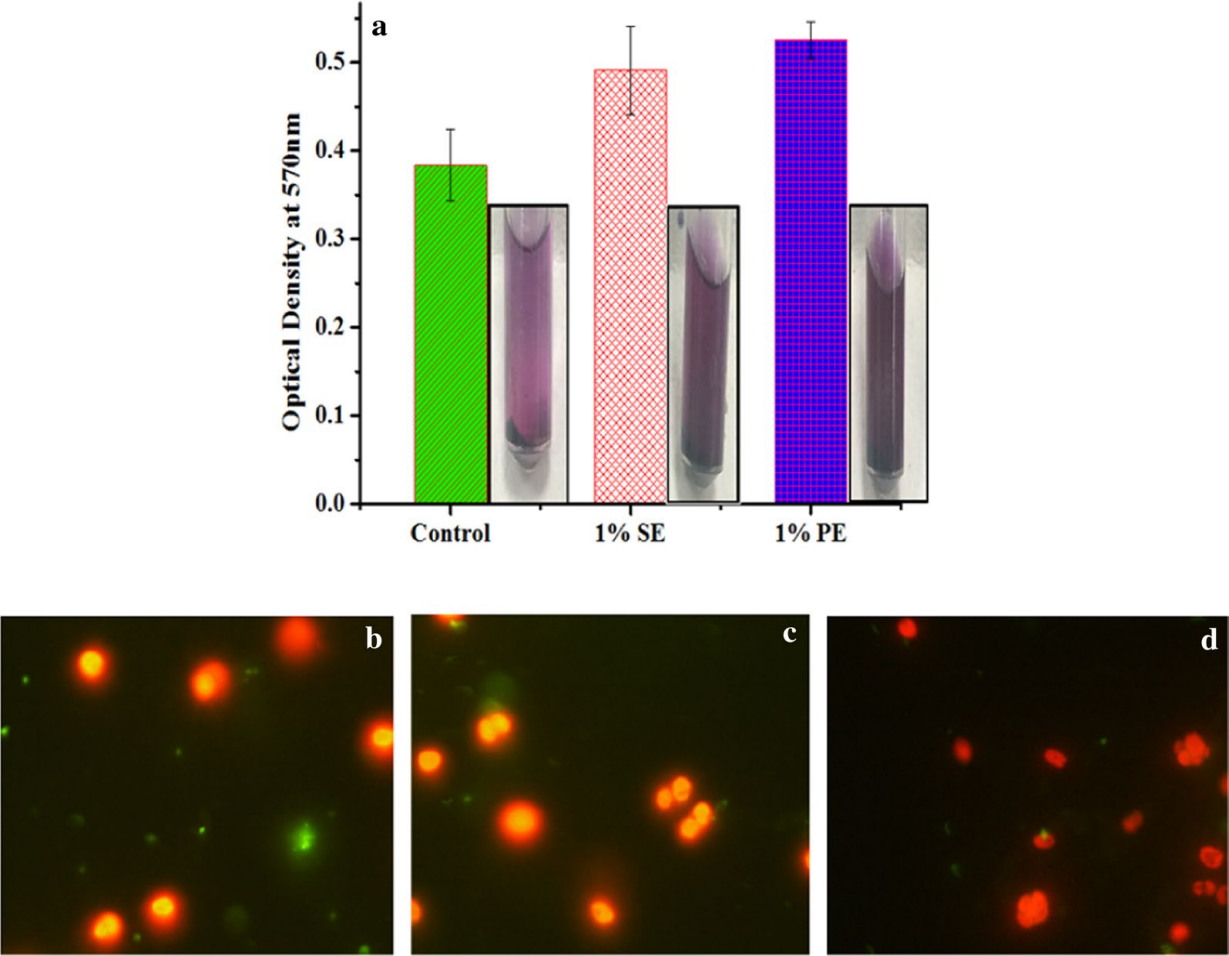
Microalgal cell viability analysis using MTT assay and SYTOX ^®^ Green method. **a** MTT result comparison of control (BG 11), 1% silicone oil nanoemulsion (1% SE) and 1% paraffin oil nanoemulsion (1% PE) in terms of optical density at 570 nm with corresponding vials showing purple formazan during the MTT Assay. Dark purple color represents greater cell viability. Errors bars are shown ± SD in MTT analysis. **b** SYTOX^®^ Green-stained fluorescent micrographs of *C. pyrenoidosa* to differentiate live and dead cells: **b** control (BG 11), **b2** 1% silicone oil nanoemulsion, **b3** 1% paraffin oil nanoemulsion. Live cells appear red while dead cells appear green in color

The viability of cell indicates the reproductive capacity of cells and also represents the physiological state which involves the production of enzymes like oxidoreductase, etc. [72]. In the case of microalgal and cyanobacterial cultures, tetrazolium compounds have been used for viability assessment [73]. Hence in this study, the viability testing of microalgal cells grown in 1% SE and 1% PE emulsion has been conducted on the 8th day of cultivation using tetrazolium dye, i.e., MTT via MTT assay. The sensitivity of the MTT assay is based on the ability of succinate dehydrogenase, microalgal mitochondrial enzyme, to convert MTT to a water-insoluble formazan dye in viable cells. The viability of cells is based on the intensity of purple color, which shows that darker the color, the more the viability in cells. In present study, the OD_570_ measured for microalgal cells cultivated in 1% PE was higher than 1% SE and control (Fig. 5a). This represents that microalgal cells were more viable in both the nanoemulsions compared with control. Additionally, the obtained results conclude that 1% PE and 1% SE nanoemulsions were non-toxic to the microalgal cells. Further, using SYTOX^®^ Green and fluorescence microscopy, a clear distinction between live and dead cells was observed. SYTOX^®^ Green stain is impermeable to the cell wall, but can stain the nucleic acid of cells when the cell wall has been damaged. The damaged cell wall allows the diffusion of SYTOX^®^ Green stain inside the cells with the compromised cell membrane and binds with the nucleic acid of the cells to generate green fluorescence. The observations confirmed that most of the microalgal cells cultivated in 1% SE and 1% PE had intact chloroplasts and membranes (Fig. 5b) as evident from emitted red fluorescence due to autofluorescence of inherent chlorophyll found in microalgal cells [74]. Only a small fraction of cells were stained by the green fluorescent dye SYTOX^®^ Green. On the other hand, the control culture showed relatively large patches of green emissions under a fluorescence microscope. Hence, from the results obtained from both MTT and SYTOX^®^ Green staining, it can be concluded that the maximum number of microalgal cells cultivated in nanoemulsions were healthy and viable on the 8th day of cultivation leading to higher cell density and biomass yield.

### Characterization

#### Bio-compositional analysis

The bio-compositional analysis was performed to evaluate the quantitative changes in macromolecules (protein, lipids, and carbohydrates) of harvested microalgal biomass cultivated in 1% SE and 1% PE compared to control (Table 3). It has been reported that during the stationary stage of cell growth, nutrient depletion occurs which results in a shift of carbon flow to storage compounds like carbohydrates and lipids [75]. An elevated CO_2_ concentration in microalgal cells could trigger the Calvin cycle, resulting in the conversion of available CO_2_ into biomolecule syntheses like carbohydrates and lipids. The biosynthesis pathway of lipids and carbohydrates compete for the same carbon source, but carbohydrate synthesis requires less energy than lipids [75]. The carbohydrate content of microalgal biomass recovered from 1% SE and 1% PE was ~ 18.9% and 17.2%, respectively, which was higher than control. Rosa et al. [76] reported that the delivery of CO_2_ through monoethanolamine enhances the biomass production and carbohydrate content (28.2% w/w) of *Spirulina* sp. LEB 18. Ji et al. [77] observed an increase in carbohydrate contents of *Scenedesmus obliquus* were 22.3%, 23.9%, and 23.6% at 5%, 10%, and 14.1% CO_2_, respectively. The finding suggests that an increase in the availability of CO_2_ to microalgal cells in cultivation media leads to enhanced carbohydrate synthesis inside microalgal cells [77]. However, the composition and metabolism of carbohydrates vary from species to species. Therefore, for biofuel production it is highly recommended to select microalgal species with efficient carbohydrate productivity [78].

**Table 3:** Biomass (biochemical) composition of *C. pyrenoidosa* in terms of lipid, carbohydrate, and protein content. The values were stated as the mean ± SD

Biochemical composition	Control	1% SE	1% PE
Carbohydrates (%)	13.6 ± 0.56	18.9 ± 0.28	17.2 ± 0.56
Proteins (%)	51.75 ± 0.77	53.75 ± 0.07	47.1 ± 0.14
Lipids (%)	18.05 ± 0.35	23.6 ± 0.84	26.8 ± 0.84

Additionally, there is an increase in lipid content in algal biomass obtained from 1% SE (23.60% ± 0.84) and 1% PE (26.80% ± 0.84) with respect to control (18.05% ± 0.35). Interestingly, in both the nanoemulsions, the highest lipid content was observed in microalgal biomass harvested from 1% PE. Ji et al. [77] reported that lipid contents of *Scenedesmus obliquus* were increased with an increase in CO_2_ concentration and were evaluated as 19.80%, 21.50%, and 22.80% at 5%, 10%, and 14.1% CO_2_, respectively. Wang et al. [79] also described that *Chaetoceros muelleri* showed increased lipid accumulation and biomass production on exposure to a 10% CO_2_ concentration. Similarly, Tang et al. [80] reported an increase in lipid content and microalgal growth in presence of 10% CO_2_ in *Scenedesmus obliquus* and *Chlorella pyrenoidosa*. The results obtained were in agreement with the reported studies.

In order to investigate the relationship between biomass productivity and lipid content, lipid productivity was calculated. The biomass of *Chlorella pyrenoidosa* obtained from 1% PE had the highest lipid productivity (69.6 mg L^−1^ day^−1^) followed by 1% SE harvested biomass (51.9 mg L^−1^ day^−1^) compared with BG11 grown biomass (14.4 mg L^−1^ d^−1^). The lipid productivity of C. *p*yrenoidosa was enhanced by 4.8- and 3.6-fold in 1% PE and 1% SE compared with BG11. The obtained productivity was higher than 49.1 mg L^−1^ day^−1^ lipid productivity of *Chlorella minutissima* attained by Arora et al. [81] by optimizing nitrogen and phosphorous concentration. Ordog et al. [82] observed 60 mg L^−1^ day^−1^ lipid productivity at 30 °C and 2% nitrogen in *Chlorella* sp. Jiang et al. [83] cultivated *Chlorella sorokiniana* in anaerobically digested effluent from kitchen waste with seawater and obtained lipid productivity of 19.00 mg L^−1^ day^−1^. However, lipid productivity is the strain-specific response that also depends on cultivation conditions and biomass productivity. Consequently, the variation in lipid productivity of *Chlorella* sp. might be observed in the literature. Hence from the present study, it can be concluded that nanoemulsions positively affected the lipid productivity of *Chlorella pyrenoidosa.* The obtained results showed a positive relationship between biomass productivity and lipid content.

The protein content was slightly enhanced in 1% SE cultivated microalgal cells (53.75% ± 0.07) compared to control, indicating they can be used as aquaculture feedstock, which desires protein content in the range of 35 to 60 wt% [84]. The protein content of microalgal biomass harvested from 1% PE (~ 47%) decreased compared with microalgal biomass of control (~ 51%) and 1% SE (~ 53%). The high concentration of CO_2_ affects the N metabolism of algal cells indirectly. As an outcome, the assimilation of nitrogen inside algal cells increases which creates nitrogen-limiting condition [85]. Therefore, the concentration of protein, as well as chlorophyll decreases. This claim supports our experimental results obtained by 1% PE supplemented microalgal cultivation. Enhanced lipid and carbohydrate content obtained from microalgal biomass recovered from nanoemulsions suggests better prospects in various applications.

#### FTIR analysis

**Fig. 6:**
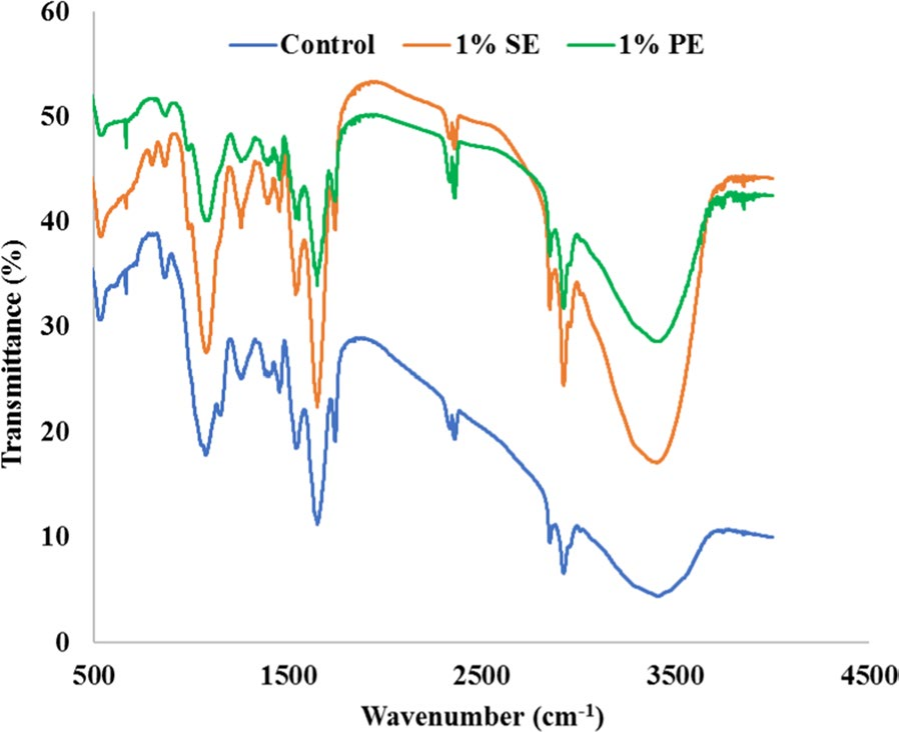
FTIR spectra of *C. pyrenoidosa* biomass obtained from control (BG 11), 1% silicone oil nanoemulsion (1% SE), and 1% paraffin oil nanoemulsion (1% PE) with different functional groups

The microalgal samples were observed using Fourier transform infrared (FTIR) technique (Table 4). The identification of peak is based on a comparison of the bands from recorded FTIR spectra of microalgae with reference literature [86]. The FTIR transmittance of the *C. pyrenoidosa* showed the presence of Si–O, P = O, C–OH, –CO, –COOH, functional groups (Fig. 6). IR spectra of *C. pyrenoidosa* showed higher intensity of lipid and carbohydrates functional groups from algal biomass of 1% SE and 1% PE. The strong peaks at 1652–1653 cm^−1^, 1266 cm^−1^, 1089 cm^−1^ representing amide I, protein, and nucleic acid proteins were observed in microalgal biomass recovered from 1% SE. The bands at 1200–950 cm^−1^ have shown absorption strength in biomass of both, i.e., 1% SE and 1% PE which directs the presence of carbohydrate content compared to control. Similarly, Ansari et al. [87] reported the presence of a strong peak in the range of 1000–1200 cm^−1^ in *Chlorella* sp*.* which depicts carbohydrate potential*.* Meng and Kassim [88] reported similar findings of an increase in band intensity in the region of carbohydrate in presence of elevated CO_2_ concentration in microalgae. The intense peak was found in the region of 1000–1150 and 2200–3000 cm^−1^, which is less evident in control cells. This represents the accumulation of carbohydrates and lipids of microalgal cells harvested from nanoemulsions. Two regions are commonly used for the assessment of lipid content. One is at 1740 cm^−1^, conveying stretching of the ester bond, and other between 2800–3000 cm^−1^ having a methyl and methylene group [89]. The strong absorption bands observed at 1456, 1744, 2852, 2922, 2924 cm^−1^ clearly shows the presence of membrane lipids and lipids representing CH_2_, CH_3_, C = O groups in biomass obtained from 1% SE and 1% PE compared with control. Correspondingly, Zawar et al. [90] described an absorption peak at 2926 cm^−1^ in *Chlorella sorokiniana* which signifies CH_2_ stretching of lipids, while peaks at ~ 1266 cm^−1^ represents carbohydrates, protein, DNA, and RNA, respectively [91]. It was clearly visible from the FTIR spectra of nanoemulsion-recovered biomass of *C. pyrenoidosa* possess fingerprints of lipids, carbohydrates, and proteins. FTIR spectroscopy appears as a viable analytical tool for revalidating the analytical data on microalgal biomass composition. The present findings are in agreement with the earlier studies for numerous microalgal species including *C. vulgaris*, *C. reinhardtii,* etc. [90, 91].

**Table 4:** Monitoring macromolecular changes in *C. pyrenoidosa* by FTIR analysis

Wavenumber range (cm^−1^)	Assignments	Functional groups	Peak (cm^−1^)
1064–880	–	Carbohydrate	965
1090–1030	P = O or Si–O	Nucleic acids	1089
1150–1000	C–O/v Si–-O	Polysaccharides/siloxane (carbohydrate peak/siloxane shoulder at 1200 cm^−1^)	1072
1263	C–O	Ester	1260
1275	C–O–H	Carbohydrates, proteins, DNA, and RNA	1266
1398–1370	CH_3_, CH_2_, C–O	Proteins, carboxylic groups	1394
1456–1450	CH_2_, CH_3_	Lipid, protein	1456
1550–1640	N–H bending	Amide	1558
1655–1638	C = O	Protein (amide I)	1652–1653
1745–1734	C = O of esters	Membrane lipids, fatty acids	1744
2875–2850	CH_2_, CH3	Lipids	2852
2930–2920	CH_2_	Lipids	2922,2924

#### Microscopic analysis

**Fig. 7:**
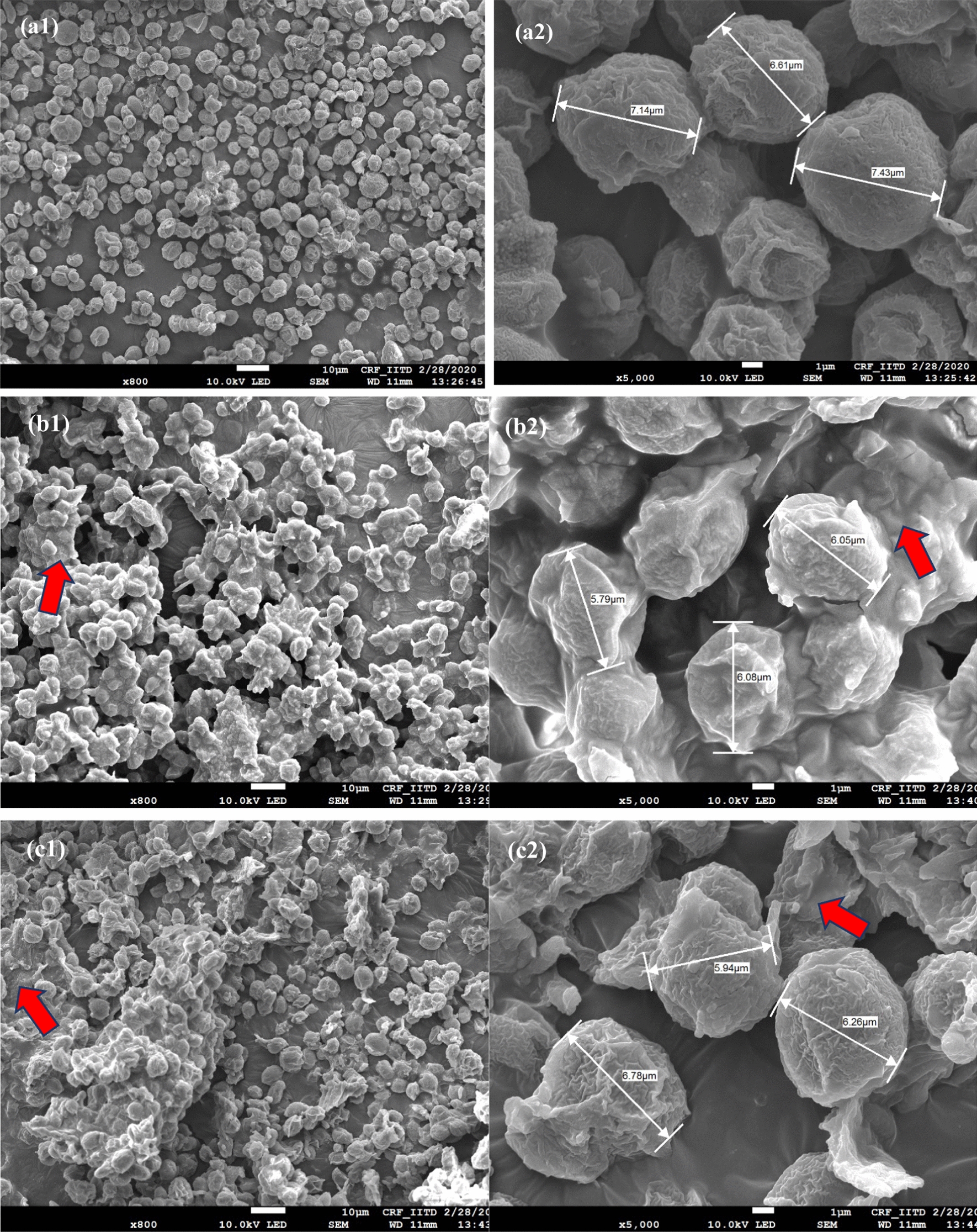
Field-emission scanning electron microscopy images of C. *pyrenoidosa*. Micrographs of BG 11 (control) cultivated *C. pyrenoidosa* at (**a1**) 800 × magnification; (**a2**) 5000 × magnification. Micrographs of 1% silicone oil nanoemulsion (1% SE) cultivated *C. pyrenoidosa* at (**b1**) 800 × magnification; (**b2**) 5000 × magnification. Micrographs of 1% paraffin oil nanoemulsion (1% PE) cultivated *C. pyrenoidosa* at (**c1**) 800 × magnification; **c2** 5000 × magnification

The biomass of *C. pyrenoidosa* obtained on day 12 was subjected to FESEM analysis to investigate the morphological variation in the cell structure. Figure 7 shows the FESEM images of *C. pyrenoidosa* biomass harvested from BG11, 1% SE, and 1% PE. From these images, it is evident that the cells were spherical and the size of the microalgal cells in control was 6 ± 2 µm which was quite similar to the cell size in 1% SE and 1% PE. In the case of nanoemulsions (Fig. 7b, c), the cells retained their basic shape, but the edges of the cell wall were not similar to cells of control. Besides, cells grown in nanoemulsions appeared morphologically intact with no cell damage. However, the microalgal cells grown in nanoemulsions were found in aggregation and displayed presence of a surface coating. This can be attributed to the release of an extracellular polymeric substance (EPS) when cells are grown in nanoemulsion. The EPS is a sticky substance released by algal cells and is composed of polysaccharides and proteins [92]. The presence of enhanced CO_2_ in 1% SE and 1% PE nanoemulsions might have triggered the microalgal cells to release this substance. It has been reported that the elevated CO_2_ concentration stimulates the metabolic carbon flux of algal cells and in order to maintain carbon balance, algae excrete organics like EPS [93]. The compositional analysis of the obtained microalgal biomass also showed that carbohydrate content was elevated in nanoemulsion-substituted cultivation which can be correlated with EPS secretions. A study suggested that secretion of EPS is known to be associated with carbohydrate content [94]. Though the present study did not quantify EPS, enhanced carbohydrate contents and aggregation could be attributed to this. As observed from the FESEM images, the intracellular adhesion behavior of microalgal cells during nanoemulsion-based cultivation opens the possibility of exploring biofilm-based systems. The intracellular adhesion and cellular colonization are the most influential parameter for biofilm formation [95]. The cellular colonization restricts temporary immobilization of cells and ensures cellular bridging which increases the cell density and promotes thickening of biofilm which might help in gaining biomass yield and value-added products [96].

### Other possible mechanisms for improved algal growth in nanoemulsions

It should be noted that the nanoemulsions used in the present study possess higher solubility of CO_2_ compared with conventional growth media. However, it was not obvious that the higher solubility of CO_2_ was the only reason for increased microalgal growth in nanoemulsion-substituted algal growth media. This was justified by performing an experiment with the addition of sodium bicarbonate (NaHCO_3_) as a carbon source for microalgal cultivation. The amount of sodium bicarbonate (~ 10 mg L^−1^) added was equivalent to the extra dissolved CO_2_ concentration (~ 5.3 mg L^−1^) in silicone oil nanoemulsion (evaluated from “Analysis of dissolved free CO_2_ concentration in nanoemulsions” section). The experimental results showed that with sodium bicarbonate addition, the microalgal growth, Chl-a content, and biomass concentration were slightly greater than the control, but far less than the silicone oil nanoemulsion (Additional file 1: Fig. S2–4). Based on the literature, several other factors could have resulted in greater microalgal biomass productivity in conjugation with nanoemulsions. Some of the possible mechanisms are summarized as follows:

- In our preliminary investigations, a higher concentration of oil (1%, 2%, 3%, 4%, and 5%) was used to observe the growth of *C. pyrenoidosa*. The high concentration of oil was supposed to enhance dissolved CO_2_ concentration in nanoemulsion, but microalgal cell concentration declined at a higher concentration of oil like 3%, 4%, 5% [27]. The results suggested that an increase in silicone oil concentration leads to a decrease in microalgal growth. Hence, the inference was that diminished microalgal cell growth was probably due to the low penetration of light in the media at a higher concentration of oil in the microalgal culture. Therefore, a lower concentration was used for the cultivation of microalgae in the present study.
- Sawdon and Peng [66] have reported the use of perfluorocarbon (PFC) to improve algal photosynthesis by decreasing dissolved oxygen in a tubular photobioreactor. The mechanism proposed by the authors was the absorption of oxygen by the PFC suspended in the culture which was produced by the algal cells during photosynthesis. In the proposed study also, the organic compounds possess higher solubility of oxygen [97] compared to water or the conventional growth media. Therefore, these organic solvents (silicone and paraffin oil) might help to decrease oxygen content in the continuous phase of nanoemulsions which indirectly promotes photosynthesis. However, more controlled experiments need to be carried out to observe the impact of reduced oxygen in the algal cultivation systems.
- Earlier studies have reported that nanosuspension can also enhance the mass transfer of solutes [98]. In a quiescent fluid, diffusion is the main mechanism for mass transfer. For the case of nanosuspensions, in addition to diffusion, due to the Brownian motion of the suspended particles/droplets local disturbance velocity fields are also generated [99]. The resultant microscale convection of the fluid can enhance the mass transfer rates of solutes in a suspension. In the present study also, the droplets of oil could be contributing to improved mass transfer of nutrients, dissolved gases, etc., from the bulk growth media to the algal cells.
- Scattering and absorption of light by the suspended particles in a suspension reduces the availability of light in a media and can lower the rate of photochemical reactions [100]. However, the nature of suspended particles is transparent and used in a small volume, we observed that the residence time of light inside the suspension can increase (manuscript under preparation). Both the oils used in the present work were used in a small volume and transparent, which suggests that there is an increase in the residence time of light inside nanoemulsions. This might improve the light availability in the nanoemulsion-supplemented media and the microalgal cells can efficiently utilize light to carry out photosynthesis.
- Paraffin components were found as a stimulant for algal growth at lower concentrations [101, 102]. In general, the lower carbon content oil phase can work as a source of carbon to the algal cells. This could be the possible reason for the phenomenal increase in microalgal growth in 1% PE along with enhanced CO_2_ availability for microalgal growth.

## Future perspectives

This research gave a new dimension to microalgae cultivation using nanoemulsion composed of two different oils. In the future, other potential biocompatible oils can be explored for increasing the biomass of microalgae. The next step would be to test it with actual sources of CO_2_ such as flue gas. Recyclability of nanoemulsions could also save cost and energy. Energy recovered by recycling the emulsion can be compared with energy given during the centrifugation of the nanoemulsion extraction process. These energy calculations can help to define a scale for nanoemulsion-based technology. Secondly, less energy-intensive nanoemulsion separation technology from microalgal biomass could help to scale this process.

Also, with the advancement of molecular techniques, metabolic pathways, and enzymes regulating carbon concentrating mechanisms of microalgal cells in nanoemulsions can be explored by the use of radiolabeled carbon. Additionally, the practical implementation and economic feasibility of proposed research must be analyzed through techno-economic analysis on a large scale. Life cycle assessment models must be used to evaluate the environmental impacts of nanoemulsion-based microalgal cultivation and biomass production.

## Conclusions

The present study proved that oil-in-water-based colloidal system offers a possibility of increasing microalgae production and volumetric productivity in batch systems without compromising the microalgae cellular structure. A significant enhancement in pigment synthesis (Chl-a) and biomass was observed. Also, a substantial increase in lipid as well as carbohydrate content was obtained during nanoemulsion-based microalgal cultivation. The biomass obtained can be utilized for biofuel production as well as organic fertilizer. The absorption of CO_2_ in emulsions is the possible mechanism by which biomass productivity was increased. Therefore, 1% SE and 1% PE nanoemulsions are projected as efficient support mediums that could significantly boost microalgal productivity. The integration of the nanoemulsions with microalgal cultivation might help in making the algal cultivation process more feasible at a large scale in the future.

## Supplementary Information

**Additional file 1: Figure S1.** Standard calibration curve of glucose: “Y” axis representing absorbance at 490 nm versus concentration of g Lucose in μg/mL on X-axis. From the above graph, we get an equation, Y = mx + c, where Y = Absorbance of sample at 490 nm, m = Mass, x = Carbohydrate concentration of sample, and c = Velocity constant. **Figure S2.** Growth profile of *C. pyrenoidosa* cultivated in sodium bicarbonate and 1% Silicone oil nanoemulsion (1% SE) compared with control (BG11) in terms of OD680. The microalgal growth was measured every 48 hours up to 12 days. The cultures were operated at 25 ± 1 °C with ~ 46.5 to 50 μmol m−2 s−1 light intensity for 12 days. The data shown are the average of two data points, and error bars represent standard deviation. **Figure S3.** Pigment synthesis of *C. pyrenoidosa* cultivated in sodium bicarbonate and 1% Silicone oil nanoemulsion (1% SE) compared with control (BG11) in terms of Chlorophyll-a (Chl-a).Chl-a estimation was performed every 48 hours up to 12 days. The cultures were operated at 25 ± 1 °C with ~ 46.5 to 50 μmol m−2 s−1 light intensity for 12 days. The data shown are the average of two data points, and error bars represent standard deviation. **Figure S4.** Growth profile of C. pyrenoidosa was observed in terms of biomass yield (g L-1) in Sodium bicarbonate, 1% Silicone oil nanoemulsion (1% SE), and control (BG11). The cultures were operated at 25 ± 1 °C with ~ 46.5 to 50 μmol m−2 s−1 light intensity for 12 days. The data shown are the average of two data points, and error bars represent standard deviation.

## Data Availability

The datasets representing the results of the proposed study are included in the manuscript.

## Abbreviations

SE: 1% Silicone oil nanoemulsion
PE: 1% Paraffin oil nanoemulsion
*C. pyrenoidosa*: *Chlorella pyrenoidosa*
BG11: Blue-green 11 media
PBS: Phosphate buffer saline
Chl-a: Chlorophyll-a
OD _680_: Optical density at 680 nm
DI: Deionized water
DCC: Dissolved CO_2_ concentration, MTT: 3-[4,5-Dimethylthiazol-2-yl]-2,5-diphenyl tetrazolium bromide
FESEM: Field-emission scanning electron microscope
FTIR: Fourier transform infrared spectroscopy
EPS: Extra-polymeric substance
