# Supplementary material for: A novel nanoemulsion-based microalgal growth medium for enhanced biomass production
Source: Biotechnol Biofuels. 2021 Apr 30;14:111. doi: 10.1186/s13068-021-01960-8 (PMC8091788; doi:10.1186/s13068-021-01960-8)
Supplement: Supplementary file 1 — Additional file 1: Figure S1. Standard calibration curve of glucose: “Y” axis representing absorbance at 490 nm versus concentration of g Lucose in μg/mL on X-axis. From the above graph, we get an equation, Y = mx + c, where Y = Absorbance of sample at 490 nm, m = Mass, x = Carbohydrate concentration of sample, and c = Velocity constant. Figure S2. Growth profile of C. pyrenoidosa cultivated in sodium bicarbonate and 1% Silicone oil nanoemulsion (1% SE) compared with control (BG11) in terms of OD680. The microalgal growth was measured every 48 hours up to 12 days. The cultures were operated at 25 ± 1 °C with ~ 46.5 to 50 μmol m−2 s−1 light intensity for 12 days. The data shown are the average of two data points, and error bars represent standard deviation. Figure S3. Pigment synthesis of C. pyrenoidosa cultivated in sodium bicarbonate and 1% Silicone oil nanoemulsion (1% SE) compared with control (BG11) in terms of Chlorophyll-a (Chl-a).Chl-a estimation was performed every 48 hours up to 12 days. The cultures were operated at 25 ± 1 °C with ~ 46.5 to 50 μmol m−2 s−1 light intensity for 12 days. The data shown are the average of two data points, and error bars represent standard deviation. Figure S4. Growth profile of C. pyrenoidosa was observed in terms of biomass yield (g L-1) in Sodium bicarbonate, 1% Silicone oil nanoemulsion (1% SE), and control (BG11). The cultures were operated at 25 ± 1 °C with ~ 46.5 to 50 μmol m−2 s−1 light intensity for 12 days. The data shown are the average of two data points, and error bars represent standard deviation. [file 13068_2021_1960_MOESM1_ESM.docx]

**Title: A novel nanoemulsion based microalgal growth medium for enhanced biomass production**

Harshita Nigam^1^, Anushree Malik^1*^, Vikram Singh^2*^

*^1^Applied Microbiology Laboratory, Centre for Rural Development and Technology and ^2^Complex Fluid Lab, Department of Chemical Engineering, Indian Institute of Technology Delhi, New Delhi, Hauz Khas, New Delhi 110016*, *India*

**Additional file 1**

**Figure S.1:** Standard calibration curve of glucose: “Y” axis representing absorbance at 490 nm versus concentration of g Lucose in μg/mL on X-axis. From the above graph, we get an equation, Y = mx + c, where Y = Absorbance of sample at 490 nm, m = Mass, x = Carbohydrate concentration of sample, and c = Velocity constant.

**Figure S.2:** Growth profile of C*. pyrenoidosa* cultivated in sodium bicarbonate and 1% Silicone oil nanoemulsion (1% SE) compared with control (BG11) in terms of OD_680_*_._* The microalgal growth *was* measured every 48 hours up to 12 days. The cultures were operated at 25 ± 1 °C with ~ 46.5 to 50 μmol m^−2^ s^−1^ [light intensity](https://www.sciencedirect.com/topics/earth-and-planetary-sciences/luminous-intensity) for 12 days. The data shown are the average of two data points, and error bars represent standard deviation.

**Figure S.3:** Pigment synthesis of C*. pyrenoidosa* cultivated in sodium bicarbonate and 1% Silicone oil nanoemulsion (1% SE) compared with control (BG11) in terms of Chlorophyll-a (Chl-a)*_._*Chl-a estimation *was* performed every 48 hours up to 12 days. The cultures were operated at 25 ± 1 °C with ~ 46.5 to 50 μmol m^−2^ s^−1^ [light intensity](https://www.sciencedirect.com/topics/earth-and-planetary-sciences/luminous-intensity) for 12 days. The data shown are the average of two data points, and error bars represent standard deviation.

**Figure S.4:** Growth profile of C. pyrenoidosa was observed in terms of biomass yield (g L^-1^) in Sodium bicarbonate, 1% Silicone oil nanoemulsion (1% SE), and control (BG11). The cultures were operated at 25 ± 1 °C with ~ 46.5 to 50 μmol m^−2^ s^−1^ [light intensity](https://www.sciencedirect.com/topics/earth-and-planetary-sciences/luminous-intensity) for 12 days. The data shown are the average of two data points, and error bars represent standard deviation.
